# Age‐specific incidence rates and risk factors for respiratory syncytial virus‐associated lower respiratory tract illness in cohort children under 5 years old in the Philippines

**DOI:** 10.1111/irv.12639

**Published:** 2019-03-19

**Authors:** Fumihiko Ueno, Raita Tamaki, Mayuko Saito, Michiko Okamoto, Mariko Saito‐Obata, Taro Kamigaki, Akira Suzuki, Edelwisa Segubre‐Mercado, Hananiah D. Aloyon, Veronica Tallo, Socorro P. Lupisan, Hitoshi Oshitani, RSV Working Group in the Philippines, Jhoys M. Landicho, Mark Donald C. Reñosa, Marianette T. Inobaya, Portia P. Alday, Amado O. Tandoc

**Affiliations:** ^1^ Department of Virology Tohoku University Graduate School of Medicine Sendai Japan; ^2^ Nagasaki Women’s Junior College Nagasaki Japan; ^3^ RITM‐Tohoku Collaborating Research Center on Emerging and Reemerging Infectious Diseases Muntinlupa Philippines; ^4^ Research Institute for Tropical Medicine Muntinlupa Philippines

**Keywords:** cohort study, lower respiratory tract illness, pulse oximetry, respiratory syncytial virus

## Abstract

**Background:**

Respiratory syncytial virus (RSV) is one of the main viral causes of lower respiratory tract illness (LRTI), especially in young children. RSV vaccines, including maternal and infant vaccines, are under development; however, more epidemiological studies are needed to develop effective vaccination strategies.

**Objectives:**

To estimate detailed age‐specific incidence rates and severity of RSV‐associated LRTI (RSV‐LRTI) using data from a community‐based prospective cohort study in the Philippines.

**Patients/Methods:**

Cohort children who visited health facilities due to acute respiratory symptoms were identified, and nasopharyngeal swabs were collected to detect RSV. The severity of RSV‐LRTI was assessed using the severity definition proposed by the World Health Organization. Risk factors for developing RSV‐LRTI and contribution of SpO_2_ measurement were also evaluated.

**Results:**

A total of 395 RSV episodes which occurred in children aged 2‐59 months were categorised as 183 RSV‐LRTI, 72 as severe RSV‐LRTI and 29 as very severe RSV‐LRTI. Children aged 3‐5 months had the highest incidence rate of RSV‐LRTI, at 207.4 per 1000 child‐years (95% CI: 149.0‐279.5). Younger age group, place of living and low educational level of caregivers were associated with developing RSV‐LRTI. Clinical manifestations had low levels of agreement with hypoxaemia as measured by pulse oximeter.

**Conclusion:**

The highest burden of RSV was observed in young infants aged 3‐5 months, whereas the burden was also high in those aged 12‐20 months. Future vaccination strategies should consider the protection of older children, especially those aged one year, as well as young infants.

## 1 INTRODUCTION

Human respiratory syncytial virus (RSV), recently renamed as *Human orthopneumovirus* belonging to the *Pneumoviridae* family *Orthopneumovirus* genus,^1^ is one of the most important viral pathogens which causes lower respiratory tract illness (LRTI) including bronchiolitis and pneumonia, especially in infants and young children.^2^, ^3^ Respiratory syncytial virus is divided into two subgroups, A and B, based on antigenic and sequence variations. The predominant subgroup and proportion of each subgroup vary between epidemics.^4^ A recent global estimate showed that the incidence of RSV‐associated acute lower respiratory infection was 33.1 million, with 3.2 million hospital admissions and 59 600 in‐hospital deaths in children younger than 5 years old in 2015.^5^ Despite the burden of this disease, there are no commercially available vaccines and the monoclonal antibody is only a specific measure taken to prevent severe disease in high‐risk infants.^6^, ^7^ The incidence rate (IR) of RSV‐associated lower respiratory illness (RSV‐LRTI) is higher in the first 6 months of life compared with older age groups, and the illness is generally more severe in young infants.^5^, ^8^ For these reasons, it is believed that natural maternal immunity is not enough to prevent severe RSV‐LRTI in early infancy. Different strategies for vaccination, including maternal vaccination and infant vaccination, have been proposed to prevent severe infection.^9^, ^10^ However, effective implementation of these strategies depends on understanding the epidemiological patterns of severe infections, including age‐specific incidences.

Respiratory syncytial virus infections are associated with various levels of respiratory illnesses, from cold‐like mild illness to very severe and even life‐threatening LRTI. To evaluate the disease burden and effectiveness of vaccines or other interventions, it is necessary to define the severity of RSV infection.^11^ To identify severe LRTI with hypoxaemia, percutaneous arterial oxygen saturation (SpO_2_) measurement by a pulse oximeter has been shown to be effective. Studies have reported that the recognition of hypoxaemia using a pulse oximeter improved the management of children with LRTI.^12^, ^13^ It was also shown that one of the most important indicators for severe RSV‐LRTI was an SpO_2_ level of less than 90%.^14^ Recently, the World Health Organization (WHO) expert group proposed that the measurement of SpO_2_ should be included in the severity definition for RSV‐LRTI.^11^

Several prospective cohort studies have been conducted in low‐ and middle‐income countries to define age‐specific IRs.^15^, ^16^, ^17^, ^18^, ^19^, ^20^, ^21^, ^22^ However, to date, few studies have reported the IR of RSV‐LRTI using standard definitions of severity. Hence, in this study, we analysed the data from a community‐based prospective cohort study of children younger than five years old to understand in detail the age‐specific incidence of RSV‐LRTI, using the definitions proposed by the WHO expert group.^11^ We examined possible risk factors for developing RSV‐LRTI and severe RSV‐LRTI and compared clinical manifestations between severe and non‐severe cases using data from a community‐based prospective cohort study in the Philippines. Further, we evaluated the level of concordance between the severity of the LRTI definitions and the classification based on the integrated management of childhood illness (IMCI).^23^

## 2 MATERIALS AND METHODS

### 2.1 Study design

In the previously described prospective cohort study,^24^, ^25^ a cohort of 4012 children younger than 5 years old was followed in two municipalities, Kawayan and Caibiran, on a main island of Biliran province in the Philippines, from March 2014 to June 2016. From this larger data set, we focused on respiratory tract illness (RTI) episodes identified at health facilities over a time window of exactly 2 years, between April 2014 and March 2016. Cohort children were identified by household visits. Demographic and socioeconomic information were collected using standard questionnaire forms by the study nurses, after obtaining written informed consent for participation from parents or guardians. Newborn infants were also recruited for the cohort. Duration of exclusive breastfeeding was checked with the caregivers through a retrospective interview. Study nurses recommended the caregivers to take their children to health facilities when children had difficulty breathing or chest indrawing. Those children who visited health facilities due to respiratory symptoms were evaluated by study nurses or a physician.^24^, ^25^ SpO_2_ was measured using pulse oximetry, the equipment for which (PalmSat® 2500A, Nonin Medical Inc., Minnesota, USA) was provided by the study. The study was approved by the Institutional Review Board of the Research Institute for Tropical Medicine and the Ethics Committee of Tohoku University Graduate School of Medicine.

### 2.2 Definition of RSV‐associated RTI

An RTI was defined as a respiratory episode with cough and/or difficult breathing. A new episode was identified if the onset of the new RTI episode was at least 7 days from the previous episode. An episode of RSV‐associated RTI (RSV‐RTI) was defined based on positive laboratory results for RSV. If there were two RSV‐positive samples within a month, the episode with the latter positive sample was considered as prolonged shedding, except if the RSV subgroups or the partial G gene sequences were different from each other.

### 2.3 Classification of RTI severity

We categorised RTIs into LRTI, severe LRTI and very severe LRTI based on the severity definitions proposed by the WHO expert group (Supporting Information Table S1).^11^, ^26^ Briefly, LRTI was defined as RTI with fast breathing (≥50 breaths/min in children aged 2‐11 months, ≥40 breaths/min in children aged 12‐59 months) or SpO_2_ < 95%; severe LRTI as LRTI with chest indrawing or SpO_2_ < 93% and very severe LRTI as LRTI with inability to feed, sleeping most of the time, difficult to wake or SpO_2_ < 90%. When RTI occurred in children aged 0‐1 month, the severity could not be defined based on the WHO definitions mentioned above.^11^, ^26^ Therefore, for this age group, we categorised RTI with ≥60 breaths/min or SpO_2_ < 95% as LRTI and RTI with chest indrawing or SpO_2_ < 93% as severe LRTI. We also classified RSV‐associated respiratory disease using Integrated Management of Child Illness (IMCI), both with and without the revision proposed in 2014.^26^ In this revision, children with pneumonia having chest indrawing without danger signs were classified as “pneumonia” (non‐severe disease). Hospitalisation of the children with severe respiratory disease was indicated by local treating physician.

### 2.4 Viral detection and defining RSV subgroups

Nasopharyngeal swabs were collected from RTI for RSV testing as described previously,^27^ including real‐time polymerase chain reaction (PCR) to screen for RSV,^28^, ^29^ conventional PCR for subgrouping and sequencing of the second variable region of the G gene (RSV‐A: 342 bp and RSV‐B: 330 bp).^30^, ^31^ Testing for RSV and other viruses, including rhinovirus, enterovirus, human metapneumovirus, human adenovirus, parainfluenza virus and influenza virus, was conducted using primers described in Supporting Information Table S2. Details of the procedure were described elsewhere.^24^ We did not exclude RSV‐RTI with other viruses from the analysis.

### 2.5 Statistical methods

Proportion of RSV subgroups by severe levels was compared using chi‐squared test. Agreement of severity classification between IMCI^26^ and the RSV‐LRTI severe case definition and agreement of SpO_2_ < 93% and having other clinical manifestations were measured using Cohen's kappa statistic. Age‐specific IRs were calculated for every three‐month age group, except for children aged <3 months. These IRs were calculated separately for 0‐1 month (as undefined RSV‐RTI) and 2 months old. The IRs of each age group were calculated as the sum of new episodes divided by the sum of the child‐years obtained for each age group. Poisson regression model was used to calculate 95% confidential interval (CI) of IRs. To evaluate the risk factors associated with developing RSV‐LRTI, we calculated hazard ratios (HRs) for total RSV‐LRTI (including RSV‐LRTI, severe and very severe RSV‐LRTI) and total severe RSV‐LRTI (severe LRTI and very severe LRTI) using the Cox proportional hazard model. Adjusted HRs (aHRs) were calculated by adjusting for age, sex and place of living (municipality) and other factors with *P* < 0.1 in the univariate analysis. Socioeconomic status was assessed using the Simple Poverty Scorecard^®^ Poverty‐Assessment Tool Philippines.^32^ Extreme poverty was defined as a score <30 points. The hazard ratio for exclusive breastfeeding was calculated separately for children <6 months. The frequencies of the clinical manifestations were compared between children with severe (severe RSV‐LRTI and very severe RSV‐LRTI) and non‐severe (RSV‐RTI and RSV‐LRTI) cases, using logistic regression models. Adjusted odds ratios (aORs) were calculated by adjusting for age, sex and place of living. The cox proportional hazard regression models and the logistic regression models were performed using R version 3.4.4,^33^ with the survival package.^34^

## 3 RESULTS

### 3.1 Categorisation of RSV‐RTI episodes

**Figure 1:**
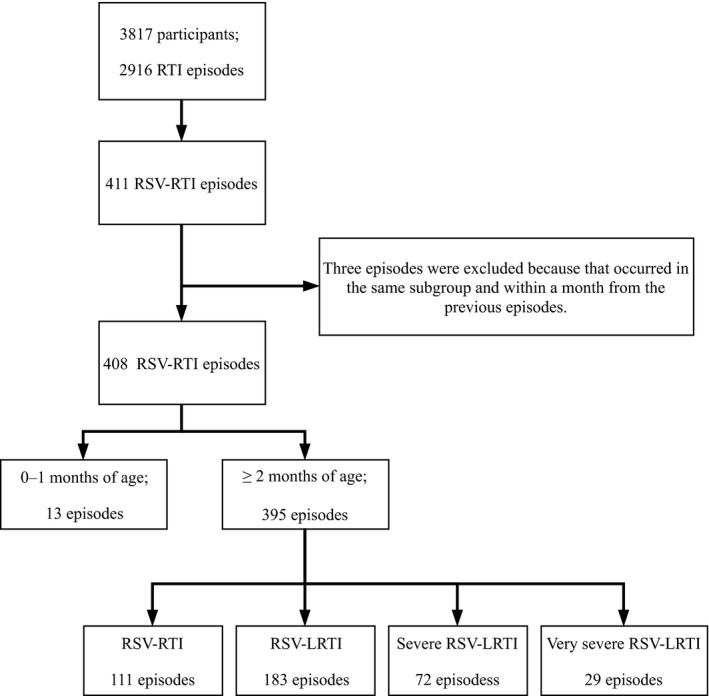
Study participants and severity assessment for respiratory syncytial virus‐associated respiratory tract illness (RSV‐RTI) in Biliran cohort study, Philippines, from April 2014 to March 2016. LRTI, lower respiratory tract illness

The first RSV epidemic was observed from May 2014 to January 2015 with the RSV mainly being subgroup A, and the second epidemic was observed from October 2015 to January 2016, with the RSV mainly being subgroup B.^27^ This analysis included 3817 children, yielding 4629 child‐years of follow‐up from April 2014 to March 2016. During this period, 2916 RTI episodes were identified at the health facilities, among which, 408 were classified as RSV‐RTI (Figure 1).

Among 408 RSV‐RTI episodes, 395 occurred in children aged 2 months or older, and 284 (71.9%) of those were placed into the RSV‐LRTI categories, including 183 RSV‐LRTI, 72 severe RSV‐LRTI and 29 very severe RSV‐LRTI (Figure 1). Six per cent of total RSV‐LRTI, 31% (31/101) of total severe RSV‐RLTI and 62% (18/29) of the very severe RSV‐LRTI episodes were identified by SpO_2_ only (Supporting Information Figure S1). Among total severe RSV‐LRTI (n = 101), 26 had SpO_2_ measured after starting oxygen treatment and at least 24% (24/101) had SpO_2_ < 90%, but only three were hospitalised. The proportion of RSV‐associated hospitalisation was 7.1% (28/395). Of these (n = 28), SpO_2_ data were available for 12 children and 25% (3/12) of those had SpO_2_ < 90%. There were 13 undefined RSV‐RTI episodes in children aged 0‐1 month, of which eight (61.5%) were hospitalised, all with chest indrawing, and three (23.1%) had SpO_2_ < 93% (Supporting Information Table S3). None of the 408 RSV‐RTI episodes was fatal.

### 3.2 Comparison between classification systems of severity

Among the 395 cases of children aged 2 months or older, using the RSV‐LRTI severity definition, the median age of children with RSV‐RTI was inversely associated with severity (Table 1). However, 71% (51/72) of the severe RSV‐LRTI and 51% (15/29) of the very severe RSV‐LRTI episodes occurred in children aged one year or older. The number of total RSV‐RTI in the municipality of Caibiran was twice that of Kawayan. In RSV‐associated hospitalisation, 96.4% (27/28) were classified as severe or very severe RSV‐LRTI. One hospitalised child had chest indrawing and inability to feed; however, this child did not have either fast breathing or SpO_2_ < 95%; therefore, this case was not classified as RSV‐LRTI.

**Table 1:** Characteristics and distributions of RSV‐RTI episodes based on the RSV‐LRTI severity definition in children aged 2 to 59 mo

Characteristics	(1)	(2)	(3)	(4)	(2) + (3) + (4)	(3) + (4)	(1) + (2) + (3) + (4)
	RSV‐RTI (n = 111)	RSV‐LRTI (n = 183)	Severe RSV‐LRTI (n = 72)	Very severe RSV‐RLTI (n = 29)	Total RSV‐LRTI (n = 284)	Total severe RSV‐LRTI (n = 101)	Total (n = 395)
Age in months, median [IQR]	25 [11, 42]	19 [11, 30]	16 [10, 23]	12 [6, 17]	18 [10, 27]	15 [9, 22]	19 [10, 31]
Age in months, n (%)							
2‐5	14 (13)	30 (16)	10 (14)	6 (21)	46 (16)	16 (16)	60 (15)
6‐11	19 (17)	17 (9)	11 (15)	8 (28)	36 (13)	19 (19)	55 (14)
12‐23	21 (19)	70 (38)	36 (50)	12 (41)	118 (42)	48 (48)	139 (35)
24‐35	21 (19)	32 (17)	4 (6)	3 (10)	39 (14)	7 (7)	60 (15)
36‐59	36 (32)	34 (19)	11 (15)	0 (0)	45 (16)	11 (11)	81 (20)
Sex, n (%)							
Male	51 (46)	90 (49)	41 (57)	20 (69)	151 (53)	61 (60)	202 (51)
Female	60 (54)	93 (51)	31 (43)	9 (31)	133 (47)	40 (40)	193 (49)
Place of living (municipality), n (%)							
Caibiran	67 (60)	132 (72)	48 (67)	16 (55)	196 (69)	64 (63)	263 (66)
Kawayan	44 (40)	51 (28)	24 (33)	13 (45)	88 (31)	37 (37)	134 (34)
Health facility, n (%)							
Outpatient	110 (99)	183 (100)	55 (76)	19 (66)	257 (90)	74 (73)	367 (93)
Hospitalisation	1 (1)	0 (0)	17 (24)	10 (34)	27 (10)	27 (27)	28 (7)
IMCI classification, n (%)							
Cough and cold	109 (98)	7 (4)	4 (6)	3 (10)	14 (5)	7 (7)	123 (31)
Pneumonia	0 (0)	173 (94)	15 (21)	9 (31)	197 (69)	24 (24)	197 (69)
Severe pneumonia or severe disease	2 (2)	3 (2)	53 (74)	17 (59)	73 (26)	70 (69)	75 (26)
Revised IMCI classification, n (%)							
Cough and cold	109 (98)	7 (4)	4 (6)	3 (10)	14 (5)	7 (7)	123 (31)
Pneumonia	0 (0)	173 (94)	68 (94)	15 (52)	256 (90)	83 (82)	256 (65)
Severe pneumonia or severe disease	2 (2)	3 (2)	0 (0)	11 (38)	14 (5)	11 (11)	16 (4)
RSV subgroup							
RSV‐A	38 (34)	42 (23)	27 (38)	8 (28)	77 (27)	35 (35)	115 (29)
RSV‐B	70 (63)	131 (72)	43 (60)	17 (59)	191 (67)	60 (59)	261 (66)
Undefined	3 (3)	10 (5)	2 (3)	4 (14)	16 (6)	6 (6)	19 (5)
Co‐detected other viruses, n (%)							
Human adenovirus	1 (1)	0 (0)	2 (3)	0 (0)	2 (1)	2 (2)	3 (1)
Rhinovirus	6 (5)	13 (7)	8 (11)	3 (10)	24 (8)	11 (11)	30 (7)
Enterovirus	0 (0)	1 (1)	0 (0)	0 (0)	1 (0)	0 (0)	1 (0)
Human metapneumovirus	0 (0)	2 (1)	0 (0)	1 (3)	3 (1)	1 (1)	3 (1)
Parainfluenza virus	2 (2)	2 (1)	0 (0)	0 (0)	2 (1)	0 (0)	4 (1)
Influenza virus	2 (2)	3 (2)	0 (0)	0 (0)	3 (1)	0 (0)	5 (1)

IMCI, integrated management of childhood illness; IQR, interquartile range; LRTI, lower respiratory tract illness; RSV, respiratory syncytial virus; RTI, respiratory tract illness.

Percentages are rounded off to integers. Total RSV‐LRTI consists of RSV‐LRTI, severe RSV‐LRTI and very severe RSV‐LRTI.

Comparing between RSV‐RTI severity definitions and IMCI classification, 98.2% (109/111) of RSV‐RTI episodes were classified as “cough or cold” by IMCI, 94.5% (173/183) of RSV‐LRTI episodes were classified as “pneumonia” and 58.6% (17/29) of very severe RSV‐LRTI cases as “severe pneumonia or very severe disease” (Table 1). The overall agreement between non‐severe (cough and cold/pneumonia; RSV‐RTI/RSV‐LRTI) and severe (severe pneumonia or severe disease; severe RSV‐LRTI/very severe RSV‐LRTI) classifications was 91.0%, and the kappa statistic was 0.74. When we applied the revised IMCI,^26^ which excluded having chest indrawing from the criteria of severe disease, none of the severe RSV‐LRTI episodes was classified as “severe pneumonia or severe disease” by IMCI (0/72) and 52% (15/29) of the very severe RSV‐LRTI cases were classified as “pneumonia” by IMCI.

Within these 395 episodes, the subgroup of RSV was identified in 376 episodes. The proportion of severe or very severe episodes was higher in the subgroup A compared with the subgroup B without statistical significance (43.8% vs 29.9%, *P = *0.06). Other viruses were also detected in 11.4% (46/395) of the episodes. Rhinovirus was the most frequently co‐detected virus (30/46) across all severity categories of RSV‐RTI (Table 1).

### 3.3 Age‐specific IRs of RSV‐LRTI

**Figure 2:**
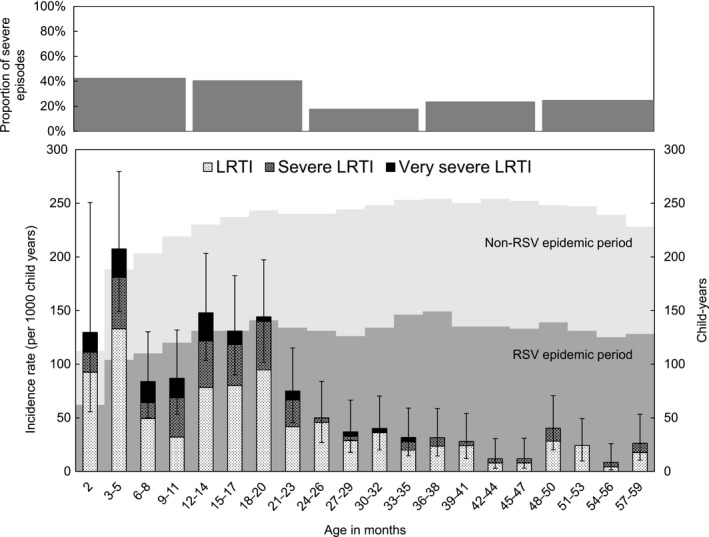
Age‐specific incidence rates of RSV‐LRTI and distributions of followed up child‐years in children aged less than 5 y in Biliran cohort study from April 2014 to March 2016. LRTI, lower respiratory tract illness; RSV, respiratory syncytial virus. The proportion of severe episodes was calculated as proportion of the total severe RSV‐LRTI episodes among total RSV‐LRTI by every 1 y of age. Incidence rates were calculated for 2 mo of age and every 3 mo of ages from 3 to 59 mo of ages. Child‐years were calculated for every 3 mo of ages. The child‐years during RSV epidemic period is shown as dark grey in background, and light grey shows child‐years out of RSV epidemic period. The epidemic periods were defined as May 2014‐January 2015 and October 2015‐January 2016 during the study period of April 2014‐March 2016. Whiskers show 95% confidence interval of incidence rate for whole RSV‐LRTI in each age group

The overall IRs of total RSV‐LRTI and total severe RSV‐LRTI were 62.1 and 22.1 per 1000 child‐years in children aged 2‐59 months, respectively (Figure 2, Supporting Information Table S4). Incidence rates for children aged 2‐23 months were 124.0 and 51.5 per 1000 child‐years for total RSV‐LRTI and total severe RSV‐LRTI, respectively, and these IRs were significantly higher than those of children aged 24‐59 months (*P* < 0.001). Children aged 3‐5 months had the highest IRs for total RSV‐LRTI (207.4 per 1000 child‐years) and total severe RSV‐LRTI (74.5 per 1000 child‐years). The IRs of total RSV‐LRTI and total severe RSV‐LRTI declined in children aged 6‐8 months compared with children aged 3‐5 months (*P* = 0.001 and *P* = 0.125, respectively). Total RSV‐LRTI increased again in children aged 12‐14 months compared with children aged 9‐11 months without statistical significance (*P* = 0.07). After 20 months old, IRs declined with age. The same trends were seen when IRs were calculated by RSV subgroup, municipality or epidemics (Supporting Information Figure S2). When we considered RSV‐RTI with ≥60 breaths/min or SpO_2_ < 95% as LRTI and RSV‐RTI with chest indrawing or SpO_2_ < 93% as severe LRTI for children aged 0‐1 month, 8 out of 13 were severe RSV‐LRTI and the overall incidence rate of total RSV‐LRTI and that of total severe RSV‐LRTI were 63.1 and 23.5 per 1000 child‐years in children aged 0‐59 months, respectively.

Fast breathing is one of the criteria for LRTI, and the cut‐off points differ by age (50 breaths/min in children aged 2‐11 months and 40 breaths/min in children aged 12‐59 months). To exclude the influence of age on the criteria for fast breathing, we calculated age‐specific IRs of RSV‐RTI with chest indrawing or SpO_2_ < 93% independently without using fast breathing. Incidence rates of RSV‐RTI episodes with chest indrawing and SpO_2_ < 93% were still high in those aged 12‐20 months, and particularly IR in 12‐14 months was higher than those in 6‐8 months for both chest indrawing and SpO_2_ < 93%. (Supporting Information Figure S3). The proportion of the total severe RSV‐LRTI episodes among total RSV‐LRTI was 42.7% (35/82) in the first year of life (2‐11 months) with the highest proportion in 9‐11 months (Figure 2). The proportion did not decline in the second year of life (40.7%, 48/118, *P = *0.777) and decreased significantly only in the third year of life (17.9%, 7/39, *P = *0.010).

### 3.4 Risk factor analysis for developing total RSV‐LRTI and total severe RSV‐LRTI

In the univariate analysis, younger age group, place of living and educational level of caregiver were significantly associated with developing both total RSV‐LRTI and total severe RSV‐LRTI (Table 2). Birth order and having other children aged <6 years in the same household were significantly associated with developing total severe RSV‐LRTI. After the adjustment, younger age group was still significantly associated with developing both total RSV‐LRTI (range of aHR: 1.7‐8.5) and total severe RSV‐LRTI (range of aHR: 7.5‐11.3) compared with the age group of 36‐59 months (Table 2). Living in the municipality of Caibiran was significantly associated with both total RSV‐LRTI (aHR: 2.5, 95% CI: 2.0‐3.2, *P* < 0.001) and total severe RSV‐LRTI (aHR: 1.9, 95% CI: 1.3‐2.8, *P* = 0.002)*. *Low educational level of caregiver (≤10 years of education) was also a significant risk of developing total RSV‐LRTI (range of aHR: 1.5‐1.7). The aHR was lower in the group with exclusive breastfeeding than those with non‐exclusive breastfeeding, but this difference was not statistically significant (aHR: 0.2, 95% CI: 0.0‐1.2, *P* = 0.09). The other factors we examined were not significantly associated with developing RSV‐LRTI.

**Table 2:** Hazard ratios of risk factors for developing total RSV‐LRTI and total severe RSV‐LRTI in children aged 2‐59 mo

Risk factors	Child‐years (n = 4571)	Total RSV‐LRTI (n = 284)						Total severe RSV‐LRTI (n = 101)					
		n	IR	HR	95% CI	aHR^a^	95% CI	n	IR	HR	95% CI	aHR^b^	95% CI
Age in months													
2‐5	242	46	190.1	8.3**	5.6‐12.4	8.5**	5.7‐12.7	16	66.1	11.8**	5.5‐25.4	11.3**	5.2‐24.3
6‐11	421	36	85.5	3.7**	2.4‐5.8	3.8**	2.5‐5.9	19	45.1	8.1**	3.9‐16.9	7.5**	3.6‐16.0
12‐23	950	118	124.2	5.4**	3.9‐7.6	5.4**	3.8‐7.5	48	50.5	9.1**	4.7‐17.4	8.3**	4.3‐16.1
24‐35	986	39	39.6	1.7**	1.1‐2.7	1.7**	1.1‐2.7	7	7.1	1.3	0.5‐3.3	1.2	0.5‐3.2
36‐59	1972	45	22.8		Ref		Ref	11	5.6		Ref		Ref
Sex													
Male	2395	150	62.6		Ref		Ref	60	25.1		Ref		Ref
Female	2177	134	61.6	1.0	0.8‐1.2	1.0	0.8‐1.3	41	18.8	0.8	0.5‐1.1	0.8	0.5‐1.2
Place of living (municipality)													
Caibiran	2168	196	90.4	2.5**	1.9‐3.2	2.5**	2.0‐3.2	64	29.5	1.9**	1.3‐2.9	1.9**	1.3‐2.8
Kawayan	2404	88	36.6		Ref		Ref	37	15.4		Ref		Ref
Gestational age													
≥36 wk	4145	268	64.7		Ref		Ref	97	23.4		Ref		Ref
<36 wk	51	3	58.8	0.9	0.3‐2.7	1.0	0.3‐3.0	2	39.2	1.7	0.4‐6.5	1.8	0.4‐7.2
Unknown	375	13	34.7	0.5**	0.3‐0.9	0.8	0.4‐1.5	2	5.3	0.2**	0.1‐0.9	0.4	0.1‐1.8
Birthweight (g)													
≥ 2500	3053	204	66.8		Ref		Ref	71	23.3		Ref		Ref
<2500	518	30	57.9	0.9	0.6‐1.3	0.9	0.6‐1.4	12	23.2	1.0	0.5‐1.8	1.1	0.6‐2.0
Unknown	1000	50	50.0	0.7*	0.5‐1.0	1.0	0.7‐1.4	18	18.0	0.8	0.5‐1.3	1.3	0.8‐2.3
No. of family member (persons)													
<7	2385	139	58.3		Ref		Ref	51	21.4		Ref		Ref
≥7	2187	145	66.3	1.1	0.9‐1.4	1.1	0.8‐1.3	50	22.9	1.1	0.7‐1.6	0.8	0.5‐1.2
Having smoker in the same HH													
No	1916	122	63.7		Ref		Ref	46	24.0		Ref		Ref
Yes	2656	162	61.0	1.0	0.8‐1.2	0.9	0.7‐1.1	55	20.7	0.9	0.6‐1.3	0.7	0.5‐1.1
Birth order													
1st	1489	89	59.8		Ref		Ref	27	18.1		Ref		Ref
2nd or 3rd	1821	105	57.7	1.0	0.7‐1.3	0.9	0.7‐1.2	36	19.8	1.1	0.7‐1.8	1.1	0.6‐1.9
>3rd	1262	90	71.3	1.2	0.9‐1.6	1.0	0.7‐1.3	38	30.1	1.7**	1.0‐2.7	1.5	0.9‐2.7
Socioeconomic status (points)													
≥30	2309	139	60.2		Ref		Ref	49	21.2		Ref		Ref
<30	2262	145	64.1	1.1	0.8‐1.3	1.0	0.8‐1.3	52	23.0	1.1	0.7‐1.6	0.9	0.6‐1.4
Educational level of caregiver (y)													
≤6	1237	68	55.0	1.3	0.8‐1.9	1.5**	1.0‐2.3	25	20.2	1.4	0.7‐2.8	1.3	0.6‐2.8
7‐9	1320	91	68.9	1.6**	1.1‐2.3	1.7**	1.2‐2.5	25	18.9	1.3	0.7‐2.6	1.2	0.6‐2.4
10	1172	88	75.1	1.7**	1.2‐2.5	1.7**	1.2‐2.5	39	33.3	2.3**	1.2‐4.4	2.1**	1.1‐4.1
≥11	843	37	43.9		Ref		Ref	12	14.2		Ref		Ref
Educational level of father (y)													
≤6	1342	79	58.9	0.9	0.6‐1.3	0.8	0.5‐1.1	37	27.6	2.4*	1.0‐6.1	2.2	0.8‐5.7
7‐9	748	49	65.5	1.0	0.6‐1.5	0.8	0.5‐1.3	16	21.4	1.9	0.7‐5.0	1.7	0.6‐4.8
10	502	25	49.8	0.8	0.4‐1.3	0.6*	0.4‐1.0	8	15.9	1.4	0.5‐4.2	1.2	0.4‐3.6
≥11	437	29	66.4		Ref		Ref	5	11.4		Ref		Ref
Unknown	1543	102	66.1	1.0	0.7‐1.5	0.9	0.6‐1.3	35	22.7	2.0	0.8‐5.0	2.0	0.7‐5.3
House wall material													
Light material	1436	99	68.9		Ref		Ref	38	26.5		Ref		Ref
Strong material	3136	185	59.0	0.9	0.7‐1.1	1.0	0.8‐1.3	63	20.1	0.8	0.5‐1.1	0.9	0.6‐1.3
Kitchen location													
Outside of household	1583	99	62.5		Ref		Ref	38	24.0		Ref		Ref
Inside of household	2988	185	61.9	1.0	0.8‐1.3	0.9	0.7‐1.2	63	21.1	0.9	0.6‐1.3	0.8	0.6‐1.2
Fuel type for cooking													
Electricity, LPG or kerosene	308	17	55.2		Ref		Ref	5	16.2		Ref		Ref
Solid fuel	4263	267	62.6	1.1	0.7‐1.9	1.0	0.6‐1.8	96	22.5	1.4	0.6‐3.4	1.1	0.4‐2.9
Treatment of drinking water													
Nothing	2070	136	65.7		Ref		Ref	44	21.3		Ref		Ref
Boil or bleach	809	50	61.8	0.9	0.7‐1.3	1.1	0.8‐1.4	22	27.2	1.3	0.8‐2.1	1.3	0.8‐2.2
Buying filtrated water	1675	98	58.5	0.9	0.7‐1.2	1.0	0.8‐1.3	35	20.9	1.0	0.6‐1.5	1.0	0.7‐1.6
Others	17	0	0.0		NA		NA	0	0.0		NA		NA
Owning private toilet													
No	1983	115	58.0		Ref		Ref	40	20.2		Ref		Ref
Yes	2589	169	65.3	1.1	0.9‐1.4	1.3*	1.0‐1.6	61	23.6	1.2	0.8‐1.7	1.4	0.9‐2.1
# of other children <6 yr in HH													
None	1575	87	55.2		Ref		Ref	28	17.8		Ref		Ref
1	1843	133	72.2	1.3*	1.0‐1.7	1.1	0.9‐1.5	53	28.8	1.6**	1.0‐2.5	1.3	0.8‐2.2
≥ 2	1154	64	55.5	1.0	0.7‐1.4	0.8	0.6‐1.1	20	17.3	1.0	0.6‐1.7	0.7	0.4‐1.3
# of other children 6‐14 yr in HH													
None	1702	104	61.1		Ref		Ref	35	20.6		Ref		Ref
1‐2	2006	116	57.8	0.9	0.7‐1.2	1.0	0.8‐1.3	42	20.9	1.0	0.7‐1.6	1.0	0.6‐1.6
≥ 3	864	64	74.1	1.2	0.9‐1.7	1.2	0.9‐1.6	24	27.8	1.4	0.8‐2.2	1.1	0.6‐2.1
Breastfeeding status^c^													
Non‐exclusive breastfeeding	47	9	191.5		Ref		Ref	6	127.7		Ref		Ref
Exclusive breastfeeding	70	8	114.3	0.6	0.2‐1.6	0.6	0.2‐1.6	2	28.6	0.2*	0.0‐1.1	0.2*	0.0‐1.2
Unknown	126	29	230.2	1.2	0.6‐2.6	1.1	0.5‐2.3	8	63.5	0.5	0.2‐1.5	0.5	0.2‐1.3

aHR, adjusted hazard ratio; HH, household; HR, hazard ratio; IR, incidence rate; LRTI, lower respiratory tract illness; RSV, respiratory syncytial virus.

Total RSV‐LRTI consists of RSV‐LRTI, severe RSV‐LRTI and very severe RSV‐LRTI. Total severe RSV‐LRTI consists of severe RSV‐LRTI and very severe RSV‐LRTI. Caregiver is the person that takes care of the child, knows best about the child and makes decisions for the best interest of the child. Solid fuel contains wood, charcoal, paper, etc

a Adjusted for age group, sex, place of living, gestational age, birthweight, educational level of caregiver and number of other children <6 y in household.

b Adjusted for age group, sex, place of living, gestational age, birth order, educational level of caregiver and father and number of other children <6 y in household.

c Hazard ratio for breastfeeding status was adjusted for age group, sex, place of living and educational level of caregiver and calculated using subset data for 2‒6 y old.

* *P* < 0.1.

** *P* < 0.05.

### 3.5 Clinical signs and symptoms associated with severe and very severe LRTI in RSV‐RTI

In the comparison of clinical signs and symptoms between severe (severe RSV‐LRTI and very severe RSV‐LRTI) and non‐severe (RSV‐RTI and RSV‐LRTI) cases, having decreased breath sounds (aOR: 8.6, 95% CI: 1.9‐60.4), wheezing (aOR: 3.1, 95% CI: 1.9‐5.2), rales (aOR: 6.2, 95% CI: 3.5‐11.9), alar flaring (aOR: 26.7, 95% CI: 11.1‐75.3), axillary temperature ≥38°C (aOR: 2.0, 95% CI: 1.2‐3.4) and tachycardia (aOR: 2.0, 95% CI: 1.2‐3.3) were all significantly associated with severe cases after adjusting for age group, gender and place of living (Table 3). Grunting, decreasing breath sound and alar flaring had high specificity of more than 97%. The positive predictive value for severe cases was high for alar flaring (85.7%), whereas the negative predictive value was high for wheezing (80.7%) and alar flaring (81.5%). Although co‐detection of rhinovirus was not significantly associated with severe cases (aOR: 1.4, 95% CI: 0.6‐3.1, *P* = 0.390), the specificity for severe cases was as high as 93.5%. Decreasing breath sounds, wheezing, rale, alar flaring and chest indrawing were significantly associated with SpO_2_ < 93% (*P* < 0.05), without high levels of agreement (kappa statistic: 0.1‐0.3) (Table 3).

**Table 3:** Adjusted odds ratios for comparison of clinical manifestations between severe and non‐severe RSV‐LRTI and agreement between clinical manifestation and SpO_2_ < 93%

Clinical manifestation	Total	Non‐severe case	Severe case	aOR^a^	95% CI	*P*‐value	Sensitivity	Specificity	Agreement with SpO_2_ < 93%	
		RSV‐RTI and RSV‐LRTI	Severe and very severe RSV‐RTI							
	n = 395	n = 294 (%)	n = 101 (%)						%	κ
Illness history										
Convulsion										
No	391	290 (74.2)	101 (25.8)		—					
Yes	4	4 (100)	0 (0.0)		—		—	—	—	—
Pallor										
No	394	293 (74.4)	101 (25.6)		—					
Yes	1	1 (100)	0 (0.0)		—		—	—	—	—
Physical examination										
Grunting										
No	380	287 (75.5)	93 (24.5)		Ref					
Yes	15	7 (46.7)	8 (53.3)	2.9	1.0‐8.8	0.052	7.9%	97.6%	84.20%	0.1
Decreasing breath sound										
No	386	292 (75.6)	94 (24.4)		Ref					
Yes	9	2 (22.2)	7 (77.8)	8.6	1.9‐60.4	0.01	6.9%	99.3%	85.30%	0.1
Wheezing sounds										
No	281	227 (80.8)	54 (19.2)		Ref					
Yes	114	67 (58.8)	47 (41.2)	3.1	1.9‐5.2	<0.001	46.5%	77.1%	71.90%	0.2
Rales										
No	174	159 (91.4)	15 (8.6)		Ref					
Yes	221	135 (61.1)	86 (38.9)	6.2	3.5‐11.9	<0.001	85.1%	53.9%	54.60%	0.1
Alar flaring										
No	353	288 (81.6)	65 (18.4)		Ref					
Yes	42	6 (14.3)	36 (85.7)	26.7	11.1‐75.3	<0.001	35.6%	98.0%	83.90%	0.2
Chest indrawing										
No	325	293 (90.2)	32 (9.8)							
Yes	70	1 (1.4)	69 (98.6)	790.6	156.6‐14 558.7	<0.001	68.30%	99.70%	82.80%	0.3
Central cyanosis										
No	395	288 (81.6)	65 (25.6)		—					
Yes	0	6 (14.3)	36		—		—	—	—	—
Apnoea										
No	395	294 (74.4)	101 (25.6)		—					
Yes	0		—		—		—	—	—	—
Poor skin turgor										
No	395	294 (74.4)	101 (25.6)		—					
Yes	0		—		—		—	—	—	—
Vital signs										
Axillary temperature										
<38°C	300	232 (77.3)	68 (22.7)		Ref					
≥38°C	95	62 (65.3)	33 (34.7)	2	1.2‐3.4	0.008	32.7%	78.9%	71.00%	0.1
Tachycardia										
No	302	233 (77.2)	69 (22.8)		Ref					
Yes	93	61 (65.6)	32 (34.4)	2	1.2‐3.3	0.01	31.7%	79.3%	72.10%	0.1
Co‐detected virus										
Rhinovirus										
No	365	275 (75.3)	90 (24.7)		Ref					
Yes	30	19 (63.3)	11 (36.7)	1.4	0.6‐3.1	0.39	10.9%	93.5%	80.3	0

LRTI, lower respiratory tract illness; OR, odds ratio; RSV, respiratory syncytial virus; RTI, respiratory tract illness.

Non‐severe RSV‐RTI consists of RSV‐LRTI and RSV‐RTI. Tachycardia is defined by age: >160 pulse/min for younger than 12 mo, >150 pulses/min for 12‐35 mo and >140 pulse/min for 36‐59 mo.

a Adjusted for age group, sex and municipality

## 4 DISCUSSION

Using data from a prospective cohort study of rural communities in the Philippines, we examined the incidence rates and severity of RSV‐LRTI in detail. We estimated the overall IR of total RSV‐LRTI to be 62.1 per 1000 child‐years in children aged 2‐59 months. These rates are comparable to recent global estimates for IRs in low‐ to upper‐middle‐income countries, which are in the range of 41‐94 per 1000 child‐years in children aged 0‐59 months (from limited data with different methodologies).^5^ Other community‐based cohort studies conducted in low‐ and middle‐income countries have reported higher IRs for total RSV‐LRTI of up to 71‐94 per 1000 child‐years in Nigeria, Kenya and Indonesia.^15^, ^18^, ^20^ Such differences may be due to the fact that these studies conducted active case findings of children with acute respiratory symptoms, whereas we analysed children who visited health facilities, potentially underestimating IRs.

We found that age‐specific IRs were significantly higher in children younger than 2 years old than in the older age group, and the same pattern was observed for total severe LRTI. Among those, the IRs of total RSV‐LRTI and total severe RSV‐LRTI were especially high in children aged 3‐5 months. Peak IRs found in previous similar studies were 3‐5 months in Nigeria, 0‐5 months in Kenya and 6‐8 months in Indonesia.^15^, ^18^, ^20^ In the Indonesian study, the IR was lower in children aged 3‐5 months than in those aged 6‐8 months, which may have been related to different factors such as the level of maternal antibodies^35^, ^36^ or breastfeeding practice,^37^ since young infants were likely to develop RSV‐associated bronchiolitis and pneumonia.^38^ Interestingly, we found that IRs dropped in children aged 6‐8 months and 9‐11 months, then increased again in children aged 12‐14, 15‐17 and 18‐20 months (Figure 2), in a pattern similar to previous studies.^15^, ^18^, ^20^ In hospital‐based studies, a majority of RSV‐associated severe infections are seen in infants aged <1 year, especially in young infants aged <6 months.^19^, ^39^ However, our study and other prospective cohort studies have identified similar bimodal peaks in IRs of total RSV‐LRTI, that is, young infants aged <6 months and children aged 12‐23 months. We suspected that the increased IRs in children aged 12‐20 months might be due to the change in the criteria for fast breathing at 12 months old. However, the age‐specific IRs of severe RSV‐LRTI defined by only chest indrawing or SpO_2_ < 93% showed similar patterns, suggesting a true increase in incidence in the second years of life. Age‐specific IRs might also be affected by the timing of RSV epidemics since observed child‐year for each age group did not cover whole epidemic periods. Therefore, we compared the proportion of epidemic period in total observed child‐years for each age group. We found not much difference in the proportions of epidemic periods between age groups. The age‐specific pattern of IRs for RSV‐LRTI has important implications for future intervention strategies including vaccination. While current vaccination strategies focus primarily on protecting young infants, if the IRs of RSV‐LRTI (especially the more severe cases) are also high in children 12‐23 months, vaccination strategies may need to consider including a booster dose before 12 months.

In our study, 13 undefined RSV‐RTI episodes were identified in children aged <2 months due to unavailability of severity definitions.^11^, ^26^ Eight were hospitalised, and all of these had chest indrawing and three of them had SpO_2_ < 93%. Therefore, more than half of RSV‐RTI in children aged <2 months were considered to be severe. Incidence rates for those with chest drawing and SpO_2_ < 93% were highest in this age group, indicating the significant impact of RSV‐RTI in this age group. To reveal true burden and risk factors for this age group, further studies, particularly large‐scale birth cohort studies, should be conducted.

We found that younger age of children (range of aHRs for total RSV‐LRTI and total severe RSV‐LRTI: 1.7‐8.5 and 7.5‐11.3, respectively), living in Caibiran (aHRs for total RSV‐LRTI: 2.4, 95% CI: 1.9‐3.1 and for total severe RSV‐LRTI: 1.9, 95% CI: 1.3‐2.8) and lower educational level of caregivers (range of aHRs for total RSV‐LRTI: 1.5‐1.7) were significant risk factors for the development of total RSV‐LRTI and total severe RSV‐LRTI (Table 2). Caibiran had a larger epidemic of RSV, and particularly RSV‐B, in 2015.^27^ Although the reasons for such differences are unknown, epidemiological patterns of RSV epidemics including their size can vary among different locations and also between epidemics even in the same location. It is therefore important to conduct longitudinal studies in multiple locations to assess the true impact of RSV in communities. Low educational level of caregiver has been often cited as a risk factor for developing severe RSV infections,^40^, ^41^ as we found here. Other commonly identified risk factors, such as prematurity (OR: 2.0), low birthweight (OR: 1.9), being male (OR: 1.2), having siblings (OR: 1.6), household crowding (OR: 1.9) and non‐exclusive breastfeeding (OR: 2.2), identified in a meta‐analysis,^42^ were not significant risk factors in the present study. Prematurity and low birthweight were significantly associated with acute lower respiratory infection in case‐control studies conducted in the hospital settings with a large number of children with severe RSV‐LRTI in high‐income countries.^42^ In our study, which mainly included cases in primary care, we did not reach the statistical significance probably due to the insufficient number of children with low birthweight, prematurity and severe RSV‐LRTI. In other low‐ and middle‐income countries, only unpublished data of one case‐control study and one cohort study for prematurity and one cohort study for low birthweight were conducted and none of these studies showed prematurity or low birthweight as a significant risk factor for severe RSV‐LRTI.^42^

Children whose gestational age or birthweight was not reported by their mothers (categorised as “unknown”) were less likely to develop RSV‐LRTI compared with the reference group. There may be a recall bias that mothers who could not recall were more likely to have had children with normal weight and gestational age.

We included a relatively large number of children in our cohort study, but the number was still too small to assess all possible risk factors. Most other studies of risk factors have been conducted as case‐control studies, which are more suitable for evaluating risk factors that occur with low frequency, such as prematurity and low birthweight.^42^ In another prospective cohort study in Bohol, the Philippines, being male and having siblings were identified as significant risk factors,^43^ although there were some differences in the study designs. The Bohol study used RSV‐associated hospitalisation as an outcome and had twice as many participants as our study. On the other hand, a birth cohort study in Kilifi, Kenya, with fewer participants than ours,^44^ having 1‐2 siblings less than 6 years old living in the same household, was a statistically significant risk factor for developing total RSV‐LRTI. Thus, further studies are necessary to comprehensively evaluate various risk factors for developing severe RSV infections.

As a previous study showed,^45^ clinical manifestations, such as wheezing, rale, alar flaring and chest indrawing, were significantly associated with SpO_2_ < 93% in our study. However, these clinical manifestations had low level of agreement with SpO_2_. Predicting hypoxaemia by clinical manifestation seemed difficult because no sole clinical sign had high sensitivity and specificity as described elsewhere.^45^, ^46^ The combination of the clinical manifestations in Usen et al’s study in Gambia had 70% of sensitivity and 79% of specificity.^45^ Moreover, 32% of severe and 62% of very severe cases were only defined by SpO_2_ level without meeting other clinical criteria of the WHO RSV‐LRTI case definition. Therefore, it should be emphasised that the diagnostic value of SpO_2_ measurement increased as the level of severity increased. Some studies have shown that hypoxaemia is more prevalent in children with RSV‐LRTI compared with LRTI with other aetiology.^47^, ^48^ In our study, 19% (19/101) of the total severe RSV‐LRTI had SpO_2_ < 90%. However, only 16% (3/19) of those were actually hospitalised. Taken together, testing for hypoxaemia is crucial for evaluation of children with RSV‐associated RTI to identify severe cases. Home care for children with LRI should be considered with SpO_2_ measurement. However, setting a cut‐off point for application of the oxygen therapy and providing the necessary equipment are important to support health workers’ decision.

One limitation of our study was that we only included episodes observed in health facilities. Since health‐seeking behaviour can vary among age groups,^49^ we may have underestimated the IRs, particularly in older children who are less likely to visit health facilities than infants. Also, our study only included two sites from the same small island with two epidemics over two years, and thus, our results, including IRs, may not reflect the true burden. In addition, the study was not conducted as a birth cohort and most newborn infants were not recruited for the study immediately after birth. Therefore, observed child‐years in this age group were particularly small in infants aged less than 3 months.

## 5 CONCLUSION

Although young infants had the highest risk of developing severe and very severe RSV‐LRTI, there were also significant impacts on the second year of life. Future interventions, including vaccination, should consider children up to two years old as a population vulnerable to severe RSV‐LRTI. Integrated child care approaches should include the use of a pulse oximeter to evaluate the severity of LRTI, and adequate oxygen treatment should be given to severe LRTI with hypoxaemia.

## APPENDIX 1

Members of the RSV working group in the Philippines are: Jhoys M. Landicho^4^, Mark Donald C. Reñosa^4^, Marianette T. Inobaya^4^, Portia P. Alday^4^, Amado Tandoc III^4^
