# Supplementary figures and images for: Age‐specific incidence rates and risk factors for respiratory syncytial virus‐associated lower respiratory tract illness in cohort children under 5 years old in the Philippines
Source: Influenza Other Respir Viruses. 2019 Mar 19;13(4):339–53. doi: 10.1111/irv.12639 (PMC6586181; doi:10.1111/irv.12639)

## Slide 1
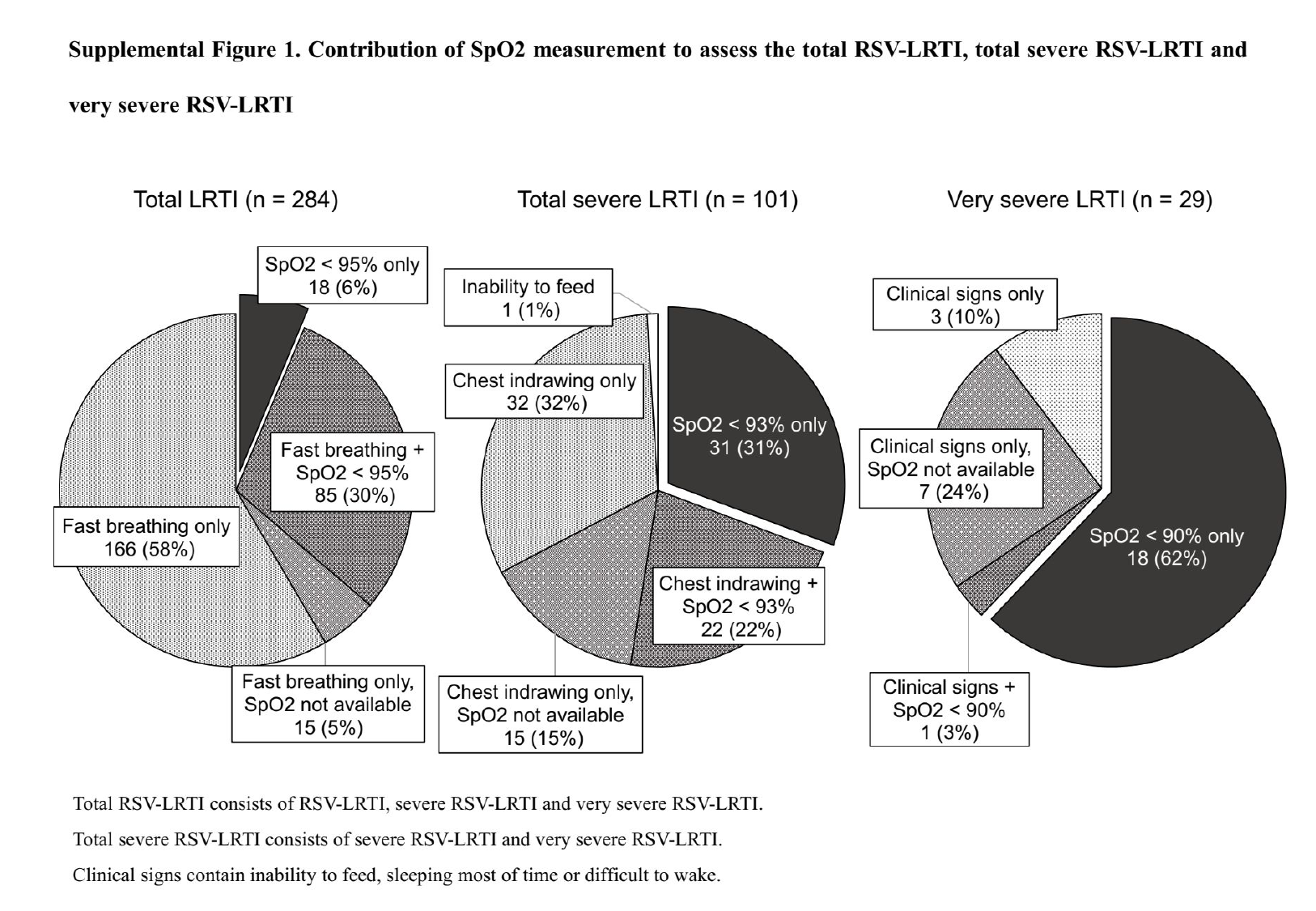

Supplement: Supplementary file 1 [file IRV-13-339-s001.pptx]

## Slide 1
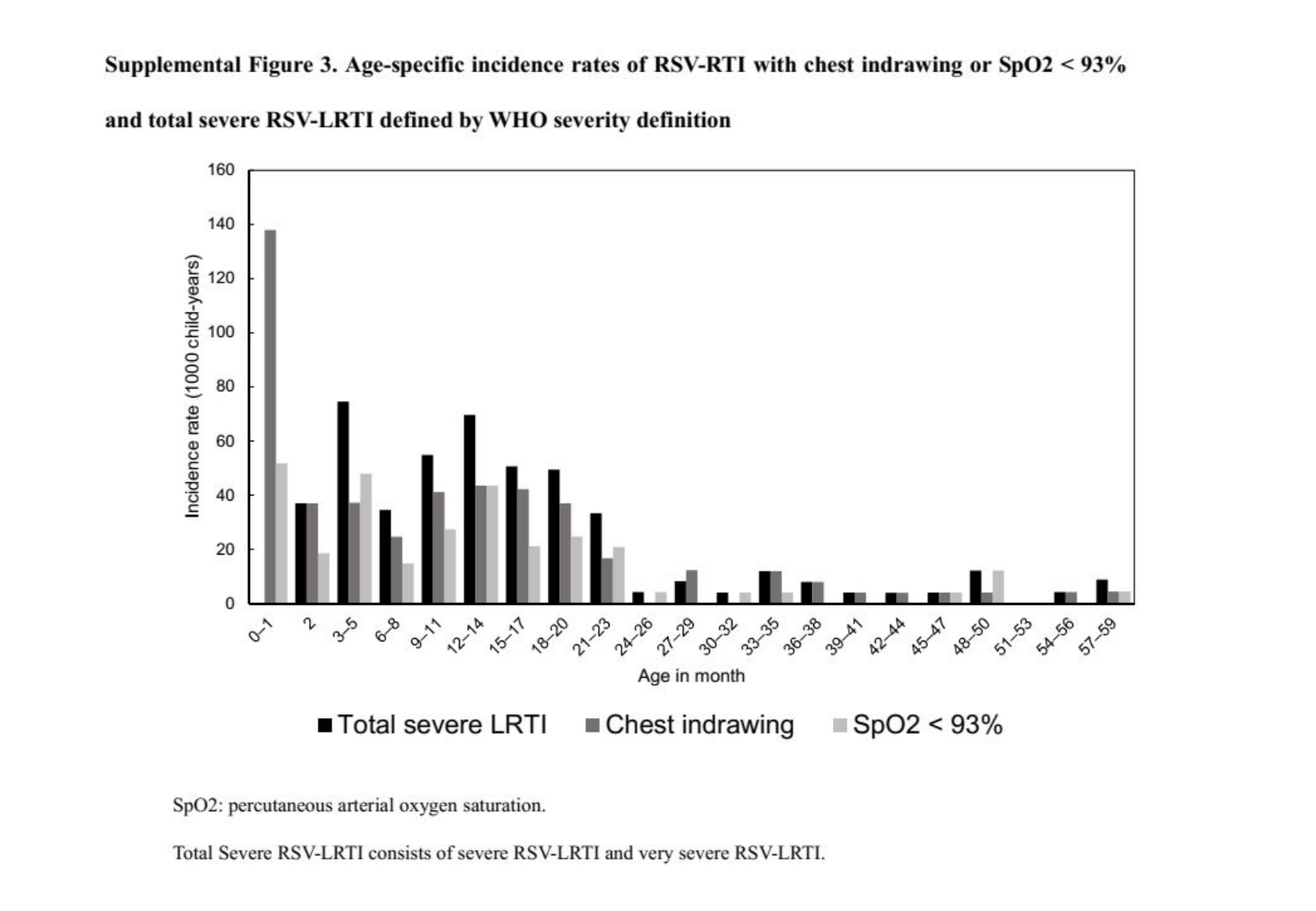

Supplement: Supplementary file 3 [file IRV-13-339-s003.pptx]
