# Supplementary material for: Age‐specific incidence rates and risk factors for respiratory syncytial virus‐associated lower respiratory tract illness in cohort children under 5 years old in the Philippines
Source: Influenza Other Respir Viruses. 2019 Mar 19;13(4):339–53. doi: 10.1111/irv.12639 (PMC6586181; doi:10.1111/irv.12639)
Supplement: Supplementary file 2 [file IRV-13-339-s002.pptx]

## Slide 1
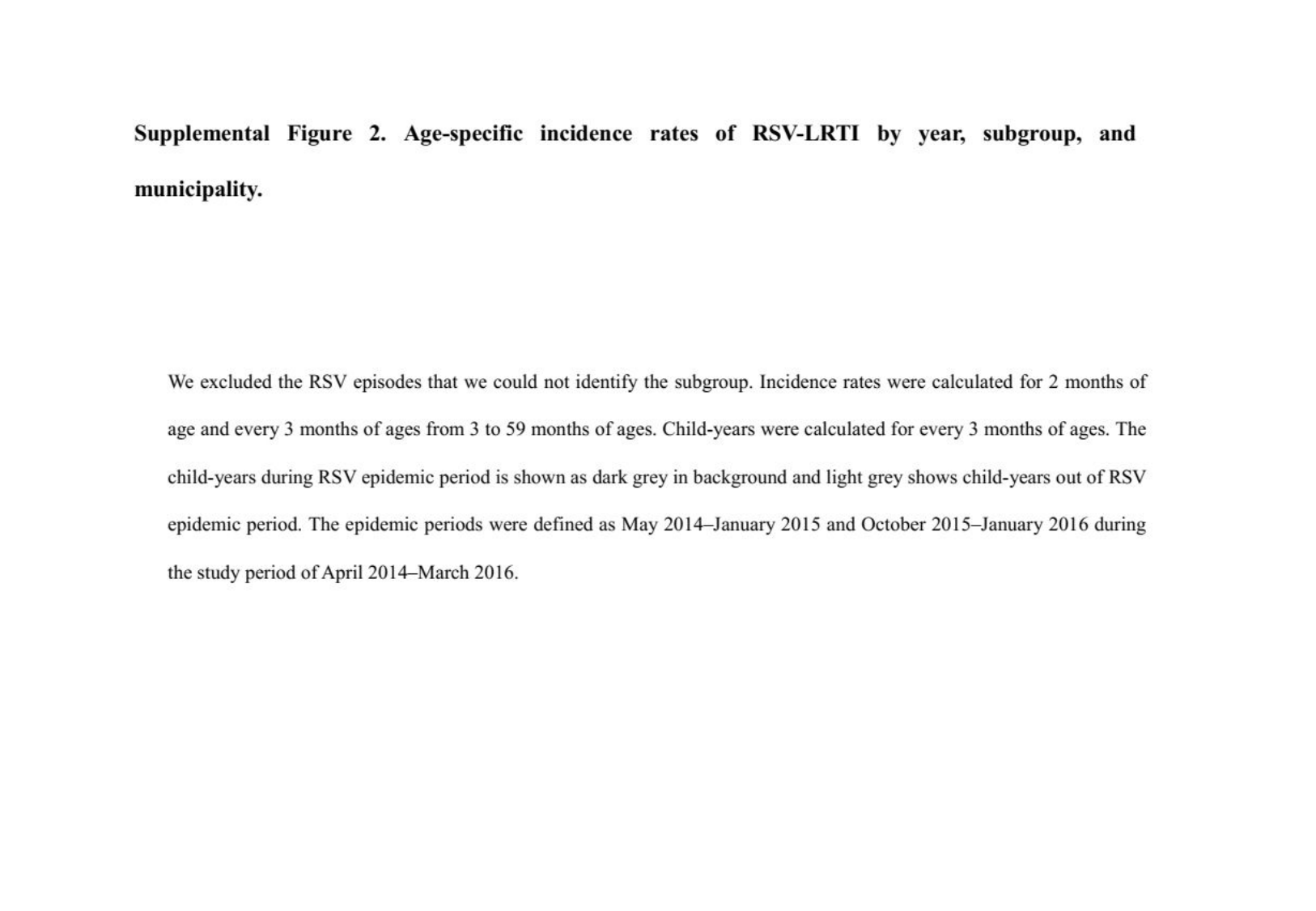

## Slide 2
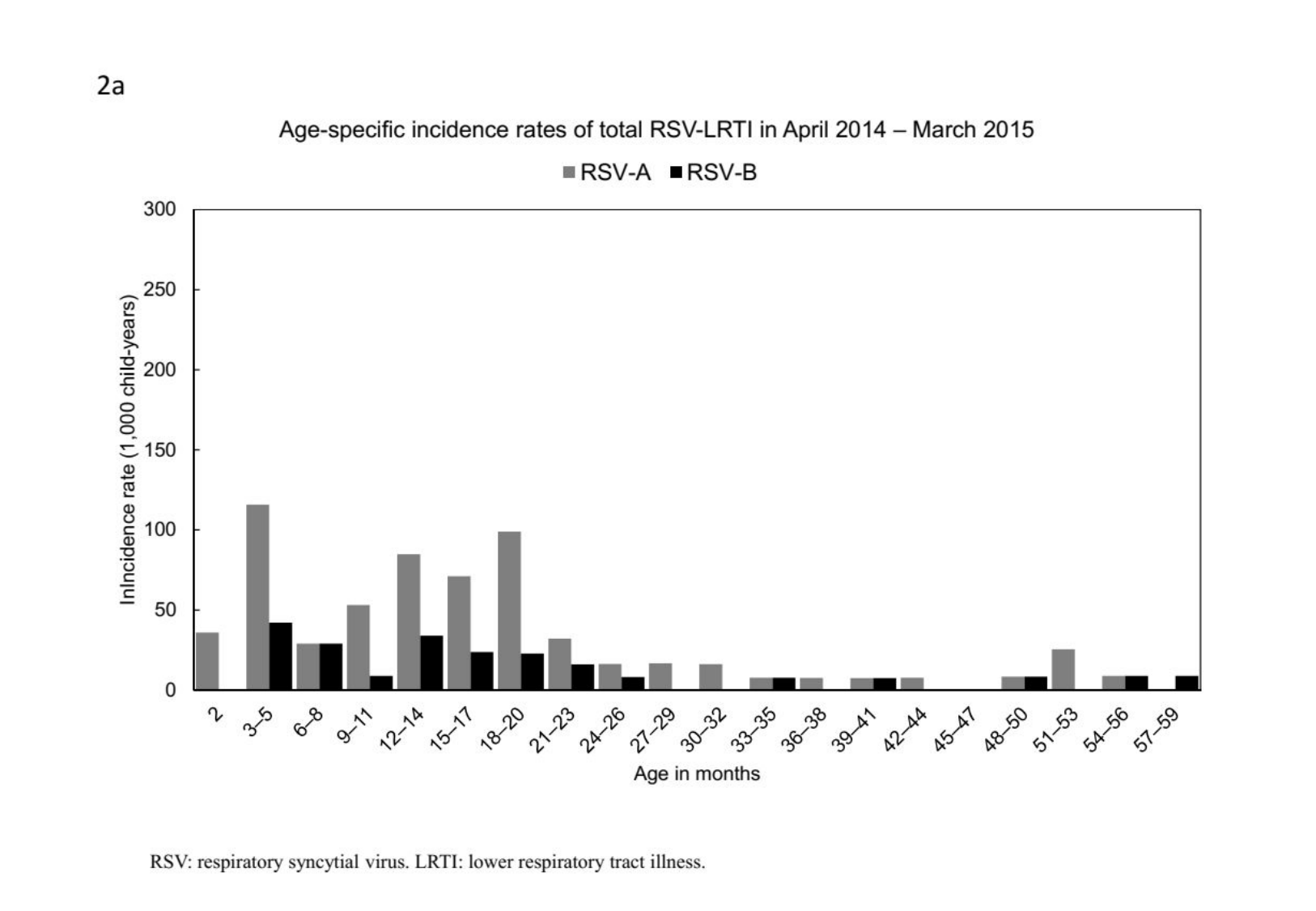

## Slide 3
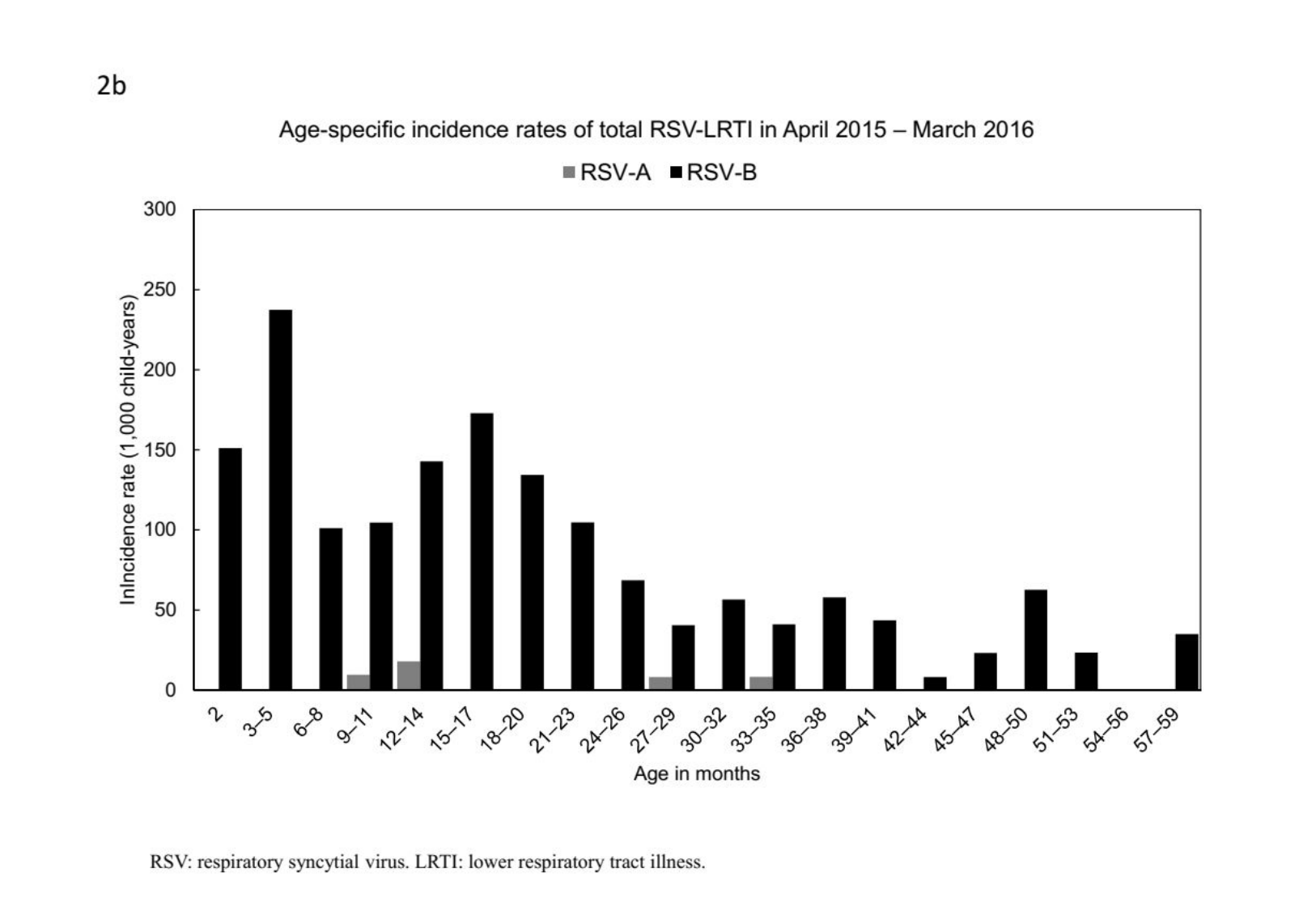

## Slide 4
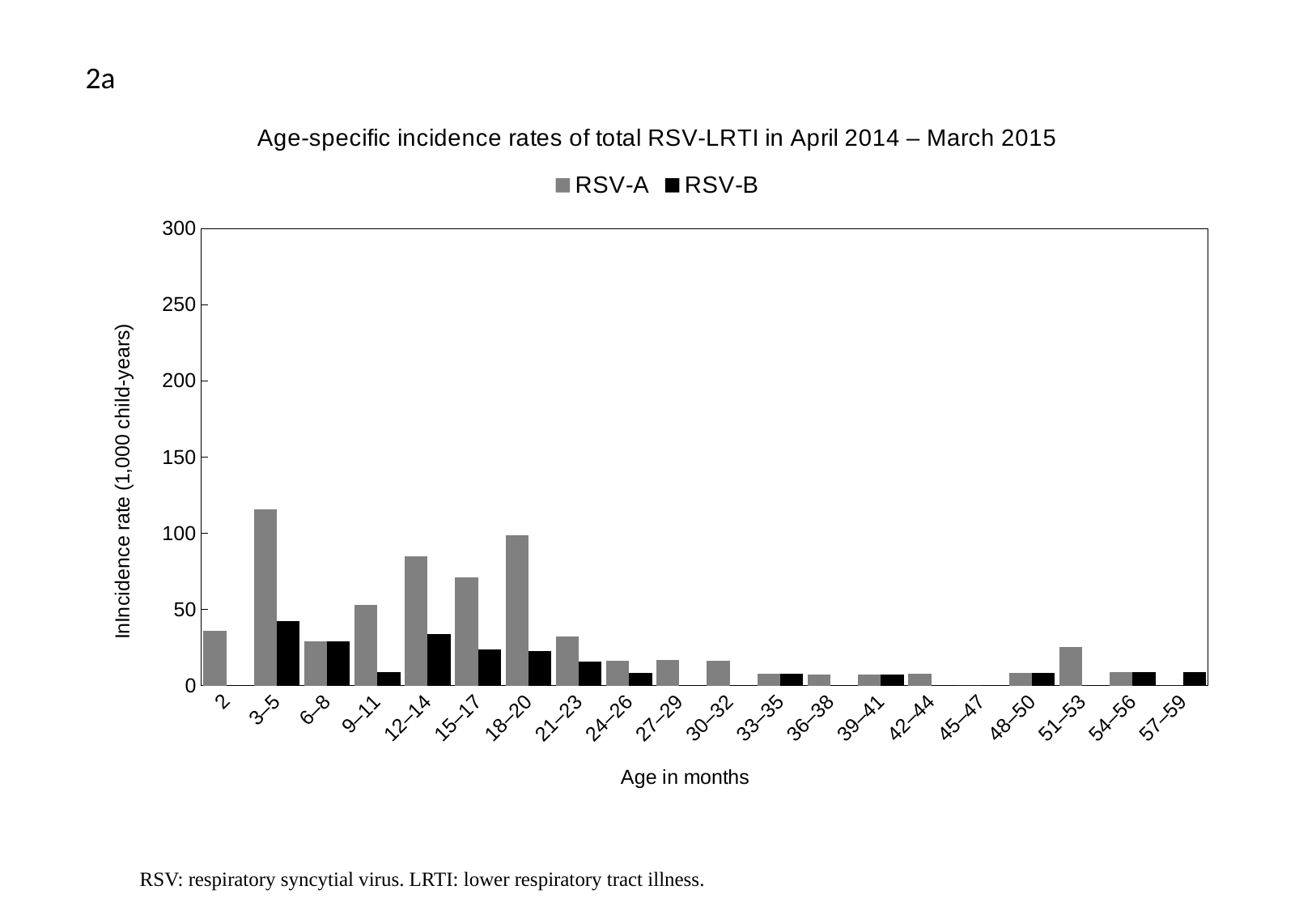

2a
### Chart: Age-specific incidence rates of total RSV-LRTI in April 2014 – March 2015
| Category | RSV-A | RSV-B |
|---|---|---|
| 2 | 35.9073928430987 | 0.0 |
| 3–5 | 115.74194105954543 | 42.08797856710743 |
| 6–8 | 29.0311042814752 | 29.0311042814752 |
| 9–11 | 53.07837628366595 | 8.84639604727766 |
| 12–14 | 84.85700346165463 | 33.94280138466185 |
| 15–17 | 71.06491990401453 | 23.68830663467151 |
| 18–20 | 98.85185493608694 | 22.81196652371237 |
| 21–23 | 32.05212583914703 | 16.026062919573516 |
| 24–26 | 16.308351751389726 | 8.154175875694863 |
| 27–29 | 16.674655892624806 | 0.0 |
| 30–32 | 16.08853650479022 | 0.0 |
| 33–35 | 7.626693950846715 | 7.626693950846715 |
| 36–38 | 7.531548993731441 | 0.0 |
| 39–41 | 7.41308274644314 | 7.41308274644314 |
| 42–44 | 7.685267011740941 | 0.0 |
| 45–47 | 0.0 | 0.0 |
| 48–50 | 8.353344768439108 | 8.353344768439108 |
| 51–53 | 25.457692486408625 | 0.0 |
| 54–56 | 8.813735189787891 | 8.813735189787891 |
| 57–59 | 0.0 | 8.787864206144889 |RSV: respiratory syncytial virus. LRTI: lower respiratory tract illness.
