# Supplementary material for: Age‐specific incidence rates and risk factors for respiratory syncytial virus‐associated lower respiratory tract illness in cohort children under 5 years old in the Philippines
Source: Influenza Other Respir Viruses. 2019 Mar 19;13(4):339–53. doi: 10.1111/irv.12639 (PMC6586181; doi:10.1111/irv.12639)
Supplement: Supplementary file 4 [file IRV-13-339-s004.docx]

**Supplemental Table 1. The category of respiratory tract illness applied in this study based on the severity definitions proposed by the WHO expert group**

| RTI | LRTI | Severe LRTI | Very severe LRTI |
| --- | --- | --- | --- |
| Cough | RTI | LRTI | LRTI |
| and/or | with | with | with |
| Difficulty breathing | Fast breathing | Chest indrawing | Inability to feed |
|  | ≥ 50 breaths/min (2–11 months of age) | and/or | and/or |
|  | ≥ 40 breaths/min (12–59 months of age) | SpO2 < 93% | Sleeping most of the time or difficult to wake |
|  | and/or |  | and/or |
|  | SpO2 < 95% |  | SpO2 < 90% |

RTI: respiratory tract illness. LRTI: lower respiratory tract illness. SpO2: percutaneous arterial oxygen saturation.

In this study, ‘sleeping most of the time’ or ‘difficult to wake’ were used as a substitution for ‘unconsciousness’ or ‘failure to respond’ described in the original definition.
