# Supplementary material for: Age‐specific incidence rates and risk factors for respiratory syncytial virus‐associated lower respiratory tract illness in cohort children under 5 years old in the Philippines
Source: Influenza Other Respir Viruses. 2019 Mar 19;13(4):339–53. doi: 10.1111/irv.12639 (PMC6586181; doi:10.1111/irv.12639)
Supplement: Supplementary file 5 [file IRV-13-339-s005.docx]

**Supplemental Table 2. Nucleotide sequences of primers and probes**

| Virus | PCR protocol | Primer/probe | 5'-3' | Gene |
| --- | --- | --- | --- | --- |
| Respiratory syncytial virus | RT-qPCR | Forward primer | GCTCTTAGCAAAGTCAAGTTRAATGATACA | N |
|  |  | Reverse primer | GTTTYTGCACATCATAATTRGGAGT |  |
|  |  | Probe | VIC-CTRTCATCCAGCAAATAYACYATCCAACGKAGYACAGG-MGB |  |
| subgroup A | RT-PCR | Forward primer | GAAGTGTTCAACTTTGTACC | G |
| subgroup B |  | Forward primer | AAGATGATTACCATTTTGAAGT |  |
|  |  | Reverse primer | CAACTCCATTGTTATTTGCC |  |
| subgroup A | Hemi-nested PCR | Forward primer | TATGCAGCAACAATCCAACC- | G |
| subgroup B |  | Forward primer | GTGGCAACAATCAACTCTGC- |  |
| Enterovirus/Rhinovirus | RT-PCR | Forward primer | CAAGCACTTCTGTTTCCC | 5'-UTR |
|  |  | Reverse primer | CACGGACACCCAAAGTAGT |  |
| Influenza A virus H1/pdm | RT-qPCR | Forward primer | AGAAAAGAATGTAACAGTAACACACTCTGT | HA |
|  |  | Reverse primer | TGTTTCCACAATGTARGACCAT |  |
|  |  | Probe | FAM-CAGCCAGCAATRTTRCATTTACC-MGB |  |
| Influenza A virus H3 | RT-qPCR | Forward primer | CTATTGGACAATAGTAAAACCGGGRGA | HA |
|  |  | Reverse primer | GTCATTGGGRATGCTTCCATTTGG |  |
|  |  | Probe | FAM-AAGTAACCCCKAGGAGCAATTAG-MGB |  |
| Influenza B virus | RT-qPCR | Forward primer | GGAGCAACCAATGCCAC | NS |
|  |  | Reverse primer | GTKTAGGCGGTCTTGACCAG |  |
|  |  | Probe | FAM-ATAAACTTTGAAGCAGGAAT-MGB |  |
| Parainfluenza virus 1 | RT-PCR | Forward primer | CCGGTAATTTCTCATACCTATG | HN |
|  |  | Reverse primer | CCTTGGAGCGGAGTTGTTAAG |  |
| Parainfluenza virus 2 | RT-PCR | Forward primer | AACAATCTGCTGCAGCATTT | HN |
|  |  | Reverse primer | ATGTCAGACAATGGGCAAAT |  |
| Parainfluenza virus 3 | RT-PCR | Forward primer | CTCGAGGTTGTCAGGATATAG | HN |
|  |  | Reverse primer | CTTTGGGAGTTGAACACAGTT |  |
| Parainfluenza virus 4 | RT-PCR | Forward primer | CCTGAACGGTTGCAYTCAGG | P |
|  |  | Reverse primer | TTGCATCAAGAATGAGTCCT |  |
| Human metapneumovirus | RT-qPCR | Forward primer | CATATAAGCATGCTATATTAAAAGAGTCTCA | N |
|  |  | Reverse primer | CCTATYTCTGCAGCATATTTGTAATCA G |  |
|  |  | Probe | FAM-CAACHGCAGTRACA CYTCATCATTRCA-BHQ1 |  |
| Adenovirus | PCR | Forward primer | CAACACCTAYGASTACATGAA | Hexon |
|  |  | Reverse primer | KATGGGGTARAGCATGTT |  |

PCR: polymerase chain reaction. RT-PR: reverse transcription PCR. qPCR: qualitative PCR.

Real-time PCR primers and probe for detecting RSV were designed by Malasao et al. modifying from Bonroy et al.^28,29^ Primer sets to detect RSV subgroups were designed by Peret et al. and Sato et al.^30,31^ Probes for real-time PCR was synthesized using custom probe service of TaqMan MGB Probes (Thermo Fisher Scientific, Massachusetts, USA).
