# Supplementary material for: Age‐specific incidence rates and risk factors for respiratory syncytial virus‐associated lower respiratory tract illness in cohort children under 5 years old in the Philippines
Source: Influenza Other Respir Viruses. 2019 Mar 19;13(4):339–53. doi: 10.1111/irv.12639 (PMC6586181; doi:10.1111/irv.12639)
Supplement: Supplementary file 6 [file IRV-13-339-s006.docx]

**Supplemental Table 3. Characteristics and clinical manifestations of children who had RSV-RTI aged 0–1 month**

| **Age (days)** | **Sex** | **Date of NPS collected**  **(year/month/day)** | **RSV subgroup** | **Admission** | **SpO2 (%)** | **Respiratory rate (breaths/min)** | **Difficult breathing** | **Chest indrawing** | **Extreme poverty** | **Birth weight (g)** |
| --- | --- | --- | --- | --- | --- | --- | --- | --- | --- | --- |
| 18 | M | 2014/9/22 | Unknown | + | (90) | 48 | - | + | - | 2810 |
| 20 | M | 2014/9/5 | A | + | 94 | 50 | - | + | + | 3100 |
| 25 | F | 2014/9/3 | A | - | 97 | 50 | + | - | + | 3075 |
| 31 | M | 2015/12/1 | B | - | 99 | 44 | - | - | - | 3480 |
| 35 | F | 2014/7/11 | A | + | 91 | 48 | - | + | - | 3000 |
| 35 | M | 2014/8/28 | A | + | 100 | 60 | + | + | + | 3400 |
| 37 | F | 2014/11/6 | A | + | 98 | 52 | + | + | + | 2395 |
| 39 | F | 2014/12/11 | A | + | 96 | 62 | + | + | - | 2280 |
| 39 | M | 2014/9/29 | A | + | (91) | 50 | - | + | + | 3200 |
| 39 | F | 2014/7/22 | A | + | (99) | 42 | - | + | + | 2900 |
| 41 | M | 2016/1/6 | B | - | 98 | 51 | - | - | - | 2540 |
| 53 | F | 2015/12/11 | B | - | 98 | 40 | - | - | - | 2460 |
| 58 | F | 2014/8/27 | A | - | 96 | 46 | - | - | - | 2800 |

RSV: respiratory syncytial virus. RTI: respiratory tract illness. NPS: nasopharyngeal swab. SpO2: percutaneous arterial oxygen saturation.

Cough was observed in all episodes, while there was no episode with inability to feed or sleeping most of the time. The SpO2 values presented in parentheses were measured during or after oxygen treatment.
