# Supplementary material for: Age‐specific incidence rates and risk factors for respiratory syncytial virus‐associated lower respiratory tract illness in cohort children under 5 years old in the Philippines
Source: Influenza Other Respir Viruses. 2019 Mar 19;13(4):339–53. doi: 10.1111/irv.12639 (PMC6586181; doi:10.1111/irv.12639)
Supplement: Supplementary file 7 [file IRV-13-339-s007.docx]

**Supplemental Table 4. The age-specific incidence rates of each severity category of RSV-RTI in children aged 0 to 59 months**

|  |  | Proposed severity by WHO | | | | | | | | | |  | |
| --- | --- | --- | --- | --- | --- | --- | --- | --- | --- | --- | --- | --- | --- |
| Age group  (months) | Child-years | Total RSV-RTI | | RSV-RTI | | RSV-LRTI | | Severe  RSV-LRTI | | Very severe  RSV-LRTI | | RSV-associated  hospitalization | |
|  |  | Number of cases | IR | Number of cases | IR | Number of cases | IR | Number of cases | IR | Number of cases | IR | Number of cases | IR |
| 0–1 | 58 | 13 | 224.1 | (5) | (86.2) | — | — | (8) | (137.9) | — | — | 8 | 137.9 |
| 2–5 | 242 | 60 | 247.5 | 14 | 57.7 | 30 | 123.7 | 10 | 41.2 | 6 | 24.7 | 5 | 20.6 |
| 6–11 | 422 | 55 | 130.6 | 19 | 45.1 | 17 | 40.4 | 11 | 26.1 | 8 | 19.0 | 5 | 11.9 |
| 12–23 | 950 | 139 | 146.3 | 21 | 22.1 | 70 | 73.7 | 36 | 37.9 | 12 | 12.6 | 13 | 13.7 |
| 24–35 | 986 | 60 | 60.9 | 21 | 21.3 | 32 | 32.5 | 4 | 4.1 | 3 | 3.0 | 4 | 4.1 |
| 36–47 | 1010 | 41 | 40.6 | 20 | 19.8 | 16 | 15.8 | 5 | 4.9 | 0 | 0.0 | 1 | 1.0 |
| 48–59 | 961 | 40 | 41.6 | 16 | 16.6 | 18 | 18.7 | 6 | 6.2 | 0 | 0.0 | 0 | 0.0 |
| 2–59 | 4571 | 395 | 86.4 | 111 | 24.3 | 183 | 40.0 | 72 | 15.8 | 29 | 6.3 | 28 | 6.1 |
| 0–59 | 4629 | 408 | 88.1 | (116) | (25.1) | (183) | (39.5) | (80) | (17.3) | (29) | (6.3) | 36 | 7.8 |

RSV: respiratory syncytial virus. RTI: respiratory tract illness. LRTI: lower respiratory tract illness. IR: incidence rate presented as per 1000 child-years

Total RSV-LRTI consists of RSV-RTI, RSV-LRTI, severe RSV-LRTI, and very severe RSV-LRTI.

Values in parentheses indicate the values when we consider RTI with ≥60 breaths/min or SpO2 < 95% as LRTI and RTI with chest indrawing or SpO2 < 93% as LRTI in 0–1 month of age.
